# The stress systems in depression: a postmortem study

**DOI:** 10.3402/ejpt.v5.26521

**Published:** 2014-12-09

**Authors:** Ai-Min Bao, Dick F. Swaab

**Affiliations:** 1 Department of Neurobiology, Institute of Neuroscience, Zhejiang University School of Medicine, Hangzhou, People's Republic of China; 2 Netherlands Institute for Neuroscience, an Institute of the Royal Netherlands Academy of Arts and Sciences, Amsterdam, The Netherlands

**Keywords:** molecular mechanisms, brain, neurotransmitters, neuromodulators, genetics, HPA axis, sex steroids, oxytocin, orexin

## Abstract

After trauma, depressive disorders are among the most frequent emerging diagnoses. However, although the symptoms of depression are well characterized, the molecular mechanisms underlying this disorder are largely unknown. Factors involved in the heterogeneous pathogenesis of depression include polymorphisms in stress-related genes, gender, age, developmental history, and environmental (traumatic) stressors such as epigenetic factors. These factors may make different parts of the stress-related brain systems more vulnerable to different stressful or traumatic life events or psychological stresses, causing alterations in a network of neurotransmitters and neuromodulators including amines, amino acids, nitric oxide (NO), and neuropeptides, and finally make individuals at risk for depression. The hypothalamo–pituitary–adrenal (HPA) axis has a prominent position in this network. With the postmortem brain material obtained from the Netherlands Brain Bank, we have carried on a series of studies with the aim to elucidate the specific changes in these systems in relation to special subtypes of depression. Our final destination is to set up tailor-made treatment for depressive patients on the basis of his/her developmental history, genetic and epigenetic background, and the vulnerability in particular neurobiological systems. This presentation is a review of our findings of changes in systems of sex steroids, receptors in the hypothalamic paraventricular nucleus, corticotrophin-releasing hormone, orexin, *γ*-aminobutyric acid, and NO in the etiology of depression, in relation to HPA activity, sex differences, and suicide.

